# Multicomponent Exercise Training Combined with Nutritional Counselling Improves Physical Function, Biochemical and Anthropometric Profiles in Obese Children: A Pilot Study

**DOI:** 10.3390/nu12092723

**Published:** 2020-09-06

**Authors:** Ana Cordellat, Beatriz Padilla, Paula Grattarola, Consolación García-Lucerga, Elena Crehuá-Gaudiza, Francisco Núñez, Cecilia Martínez-Costa, Cristina Blasco-Lafarga

**Affiliations:** 1 Physical Education and Sports Department, University of Valencia, Gascó Oliag, 3, 46010 Valencia, Spain; Ana.Cordellat@uv.es; 2 Sport Performance and Physical Fitness Research Group (UIRFIDE), University of Valencia, Gascó Oliag, 3, 46010 Valencia, Spain; 3 INCLIVA Health Research Institute, Menéndez y Pelayo, 4, 46010 Valencia, Spain; beapalo@gmail.com (B.P.); apgrattarola@gmail.com (P.G.); elenacrehua@gmail.com (E.C.-G.); fnunezg@telefonica.net (F.N.); 4 Pediatric Gastroenterology and Nutrition Section, Hospital Clínico Universitario of Valencia, Blasco Ibáñez, 17, 46010 Valencia, Spain; 5 Department of Physiotherapy, University of Valencia, Gascó Oliag, 3, 46010 Valencia, Spain; Consolacion.Garcia-Lucerga@uv.es; 6 Department of Pediatrics, University of Valencia, Blasco Ibáñez, 15, 46010 Valencia, Spain

**Keywords:** body composition, cardiovascular health, health education, insulin resistance, motor competence, overweight, physical fitness

## Abstract

Aerobics or strength exercise plus diet interventions have been shown to counteract childhood obesity. However, little is known with regard to periodized multicomponent exercise interventions combined with nutritional counselling, which might be less demanding but more enjoyable and respectful of children and adolescents’ nature. In order to analyze the impact of such a multimodal approach, 18 obese children (10.8 ± 1.6 years; 63% females; z Body Mass Index 3 ± 0.4) trained for 60 min, twice weekly and were measured for body composition, biochemical parameters and physical function. We found that 16 weeks of multimodal intervention (14 of training), based on fun-type skill-learning physical activities and physical conditioning with challenging circuits and games, together with nutritional counselling, led to an attendance > 80%, with significant overall health improvement. Body composition was enhanced (*p* < 0.01 for z BMI, mid-upper-arm-circumference, waist-to-height ratio, tricipital and subscapular skinfolds, body-fat % by Slaughter equation and Dual energy X-ray absorptiometry body fat% and trunk fat%), as well as metabolic profile (LDL cholesterol, gamma-glutamyl transferase , alanine aminotransferase ; *p* < 0.05), homeostatic model assessment of insulin resistance (HOMA-IR; *p* < 0.05) and inflammatory response (C-Reactive Protein; *p* < 0.05). Physical fitness was also improved (*p* < 0.01) through better cardiovascular test scores and fundamental movement patterns (Functional Movement Screen-7, FMS-4). Tailoring multimodal supervised strategies ensured attendance, active participation and enjoyment, compensating for the lack of strict caloric restrictions and the low volume and training frequency compared to the exercise prescription guidelines for obesity. Nutritional counselling reinforced exercise benefits and turned the intervention into a powerful educational strategy. Teamwork and professionals’ specificity may also be key factors.

## 1. Introduction

Childhood obesity and overweight represent an important public health problem, mainly in developed countries [1,2]. According to the World Health Organization (WHO) [3], more than 42 million children under the age of 5 suffered in 2013 from this pandemic phenomenon defined as an excess of fat related to several comorbidities, including metabolic, endocrine, cardiovascular, gastrointestinal, pulmonary, orthopedic, neurologic and dermatologic disorders in conjunction with psychosocial difficulties [4]. The association of childhood obesity with insulin resistance has attracted special concern, considering the key link between adiposity excess and its metabolic and vascular comorbidities [5]. Moreover, childhood obesity is highly possible to persist into adulthood [6], increasing the need for interventions addressed at preventing or treating it in the pediatric population, both in childhood and adolescence. The report of the commission *Ending Childhood Obesity* (WHO) in 2016 [7] already supported the notion that lifestyle was likely to be behind the problem and highlighted the relevance of promoting physical activity and exercise/sports habits as well as healthy diet consumption among children and adolescents.

Regarding these physical exercise or psychomotor interventions, and according to the recent review of Kelley, et al. [8], they should be the most important type of intervention for the overall physiological health of this group of age, given their clinically relevant improvements in adiposity outcomes, lack of adverse events and significant enhancements in a wide number of secondary outcomes. Regular exercise, especially aerobic training, is associated with the reduction of fasting insulin and HOMA-IR (homeostatic model assessment—insulin resistance) in children and adolescents with obesity and overweight [9]. Indeed, both aerobic exercise and resistance training—when it is metabolically demanding [10,11,12]—have been shown to improve body composition in adults [2,10,13] and children [10,11,12], preventing metabolic syndrome and type 2 diabetes [2,14].

On the other hand, not only the effect of increasing the metabolic rate and physiological responses trough augmented muscular activity but also the improvement in fitness following these exercise interventions, or their combination, has a great impact on most of the health outcomes related to metabolic syndrome and overall cardiovascular disorders in obesity [10,12,14]. They include, but are not limited to, improved autonomic functioning, endothelial function, anti-inflammatory processes, blood pressure, fasting blood glucose, insulin resistance, metabolic profile and body composition [2,15,16,17]. Higher physical activity and fitness levels are related to higher prevention [14], with greater improvements associated with greater exercise intensity [8,18,19,20] or frequency of training [2,8,10,12,18,21].

However, exercise alone might not be enough to reduce weight [10,22] or to maintain or prevent the regain of weight after exercise and diet interventions in western countries [23]. Meanwhile, the benefits of multimodal strategies, involving nutritional as well as physical exercise interventions in obese children, have been reported in many reviews and meta-analyses [8,24,25,26]. Physical exercise plus diet is a good strategy, despite children needing to be specially motivated in order to continue practicing physical activity, and they do not like to follow continuous strict rules about food and time schedules [27,28]. Adherence is low when obese children have to follow classic guidelines and caloric restriction [27,28,29], as well as boring exercise proposals. In this scenario, the evidence suggests that mixed interventions that involve nutritional changes, physical activity and behavioral strategies might be beneficial to achieve small reductions in body weight in children and adolescents of all ages [30], with larger benefits in the long term [22]. Game-based and motor control interventions may also help to make it more enjoyable and suitable [31].

Due to the need to design and improve multimodal interventions to fight childhood obesity, where low adherence and lack of proper evaluation are still a problem, the present study aims to evaluate the effectiveness of a 16-week multicomponent exercise program (14 weeks of training) in combination with nutritional counselling addressed to pediatric patients with obesity. The evolution of biochemical as well as anthropometric parameters is addressed, jointly with changes in physical function and the fundamental movement patterns, which are also important outcomes to understand the intervention’s impact and future consequences. We hypothesize that only two hours weekly of periodized and enjoyable supervised exercise training, jointly with nutritional counselling, might be enough to improve body composition, lipid profile and insulin resistance, as well as physical fitness, in this population.

## 2. Materials and Methods

### 2.1. Study Design and Participants

A prospective longitudinal study was conducted in obese schoolchildren and adolescents aged 8–14 years. Twenty-two participants were recruited from the Paediatric Gastroenterology and Nutrition Unit of a tertiary hospital in Valencia, Spain between December 2016 and January 2018. Inclusion criteria were as follows: children between 8 and 14 years, derived from the hospital, with body mass index (BMI) ≥ 2 SD for age and sex and ability to participate in physical exercise with safety according to the medical prescription. Exclusion criteria were the presence of endogenous obesity, physical or mobility impairments. All the families were fully informed and gave written consent to participate in this study, approved by the ethics committee of the University of Valencia (H1449692084171). They were also advised to maintain sleep and rest, as well as prevent any changes in medication or stimulant consumption.

**Figure 1:**
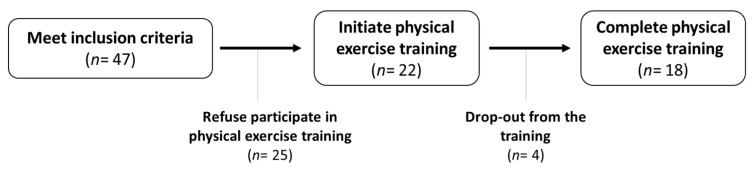
Flowchart of participants.

After an initial electrocardiogram to test the cardiac health of the participants, anthropometric, biochemical and physical function measurements were conducted to analyze the impact of 14 weeks of multicomponent exercise plus nutritional counselling. As shown in Figure 1, 18 participants completed the multimodal intervention and the whole testing program, comprising the final sample.

### 2.2. Body Composition and Biochemical Assement

Body weight (kg) and height (cm) were measured by standardized anthropometric procedures using an electronic balance (100 g precision) (Seca^®^, Hamburg, Germany) and a stadiometer (1 mm precision) (Holtain^®^, Crymych, UK). The BMI (kg/m^2^) was calculated. Nutritional status was defined based on the BMI for age z-score (z BMI) according to WHO cut-off points, considering obesity when BMI z-scores ≥ 2 SD [32]. In addition, triceps (TRI) and subscapular (SUB) skinfold thickness were measured using a skinfold caliper (0.2 mm precision) (Holtain^®^, UK). TRI and SUB measurements were taken in triplicate, and the mean was calculated for the analyses. Additionally, mid-upper arm circumference (MUAC) and waist circumference (WC) were measured to the nearest 0.1 cm with a flexible tapeline. Waist-to-height ratio (WHtR) was calculated as WC (cm)/height (cm).

Z-scores of weight for age and height for age of participants were calculated according to WHO 2007 references [32]. Z-scores for age were calculated for TRI (z TRI), SUB (z SUB), MUAC (z MUAC) and for WC (z WC), following the work of Frisancho [33] and McCarthy [34]. Additionally, percentage of body fat was estimated by Slaughter index based on skinfold measurements [35].

Dual energy X-ray absorptiometry (DXA), recognized as the gold standard for body composition assessment [36,37], was used to measure fat mass (FM) and lean mass (LM). Prior to each measurement session, the DXA equipment (Hologic, Inc., Model Discovery QDR-Series-Wi; nª 84894, Marlborough, MA, USA) was calibrated to maintain the precision and accuracy of the scanner, with a certified clinical densitometrist (CM) performing all DXA data collection and analysis. According to the Australian Institute of Sport best practice protocols for a total body scan [38], participants were motionless in a supine position on a padded exam table, while an X-ray array passed above them. LM and FM were further determined by means of the APEX 4.0, Hologic Discovery software.

The following biochemical parameters were determined in serum samples after 12 h of overnight fast according to standardized procedures: total cholesterol, HDL cholesterol (HDL-C), LDL cholesterol (LDL-C), triglycerides (TGA), uric acid, glucose, insulin, glycated hemoglobin (HbA1c), C-reactive protein (CRP), gamma-glutamyl transferase (GGT) and alanine aminotransferase (ALT). Total cholesterol, HDL-C, LDL-C, triglycerides, glucose and uric acid were measured by applying enzymatic assays using an automated analyzer (AU5400; Olympus Diagnostica GmbH, Hamburg, Germany). Additionally, insulin was determined by electrochemiluminescence with a modular autoanalyzer (Roche Diagnostics, Basel, Switzerland). The homeostatic model assessment of insulin resistance (HOMA-IR) was calculated as follows: (fasting insulin (µU/mL) × fasting glucose (mg/dL)/405).

### 2.3. Physical Function Assement

First of all, neuromuscular capacities were assessed by means of the handgrip test and the broad jump test, following Ortega, et al. [39] guidelines. Handgrip strength was first assessed (TKK 5401 Grip D; Takei, Tokyo, Japan), with the participant squeezing gradually and continuously the hand dynamometer for at least 2 s, performing the test with the right and left hands in turn. Children made two trials (alternately with both hands) interspersed with 30 s of rest, and the maximum score (in kg) was retained for each hand. Then, the lower-limb rate of force development (RFD) was tested by means of the OptoJump^TM^ infrared system (Microgate, Bolzano, Italy). Participants stood with feet approximately shoulder width apart, jumping horizontally to achieve maximum distance. They were allowed to perform a countermovement with the arms and legs before jumping horizontally. The best of three attempts was recorded (in cm).

Forty-eight hours later, neuromuscular assessment was completed with the functional movement screen (FMS), a test comprised of seven fundamental movement patterns that require a balance of mobility and stability [40,41]. All the exercises are scored on a three-point scale, with three indicating perfect performance, two indicating minor deficits or perfect performance with modifications and one indicating the inability to perform the movement [40,41]. Participants were recorded in the frontal and lateral plane for major precision, and right and left sides were registered for each exercise, using the lower value for obtaining the complete (FMS_7) or partial (FMS_4) score. This partial score (FMS_4) is extended in the literature to evaluate obese people and is based on 4 exercises from FMS battery (deep squat, hurdle step, shoulder mobility and active straight leg raise) [42] because the other 3 tests (in-line lunge, push up and rotary stability) are difficult to execute in this population [43]. Importantly, all the videos were analyzed by two evaluators, keeping an agreement percentage above 90%.

Later on, cardiovascular fitness (CVF) was assessed by means of the modified progressive aerobic capacity endurance run (mPACER), a specific test for obese children [44]. Following the mPACER guidelines, participants run from one marker to the opposite set at 20 m, starting at 4 km/h and increasing 1.5 km/h each minute until the 8.5 km/h is reached. From this speed, guidelines were based on the original PACER. The audio was pre-recorded. The test stops when the child can no longer maintain the pace, being unable to reach the end of the path during the audio signal twice. The number of laps completed was recorded, so the more laps, the better the cardiovascular fitness. Children wore a heart rate monitor (Polar RS800) to prevent cardiovascular exhaustion.

A familiarization session was conducted two days before the testing days and again on the testing day, so the rules and protocols were refreshed. The FMS served as warm-up for the mPACER, which starts at walking intensity.

### 2.4. Multimodal Intervention

Details of the exercise and nutritional habits of each participant were collected in order to personalize the intervention and obtain proper adherence. The intervention lasted 16 weeks and was composed by a periodized multicomponent exercise program in combination with nutritional counselling.

(a) Multicomponent training program

**Figure 2:**
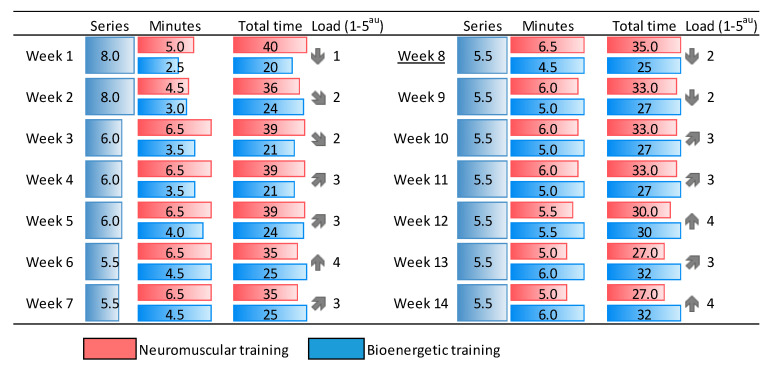
Multicomponent training program. Neuromuscular contents were prevalent in weeks 1 to 7, a trend inverted in weeks 9 to 14, when metabolic demands were increased after some controls in week 8. Load 1–5^au^ (arbitrary units) and the grey arrows in the column (formatted in excel) display the load undulation, where 5 would be the maximum exertion tolerated for the session.

As shown by Figure 2, motor time was divided into sets, interspersing neuromuscular and endurance contents in each session. In order to improve the motor literacy and, as fast as possible, increase the time of exercising at high intensity with technique and safety, the periodized program was initiated with larger neuromuscular demands and evolved to more demanding metabolic proposals, mainly from week 9. In addition, most proposals were designed with ludic and motivational schemes, altering constraints continually and combining the motor-control dual-task with games, which is especially suitable for children and adolescents. For the same reason, the methodology also evolved, initiating the program with maximal guidance instructions and changing later to autonomous methods, aiming to make the participants feel the need for movement and become self-aware of the wellbeing associated with physical exercise.

More specifically, the duration and number of sets was previously fixed in all sessions (60 min 2 days/week), alternating neuromuscular and bioenergetics contents (Figure 2). Neuromuscular exercises included strength, balance and mobility postural control exercises at the beginning and later some fighting and/or carrying games, as well as resistance training with challenging circuits. Bioenergetics comprised fun-type skill-learning physical activities, with dual-task and mini-sport games. This alternating structure allowed us to maintain the participation of large muscle mass at moderate to vigorous intensities throughout the 60 min period. The short multicomponent intervention (16 weeks, where the first and the last one were used to assess physical outcomes) was designed and conducted by sports sciences graduates.

Motor time sequences and intensities undulated throughout the 14 weeks, besides changing from the initial 20 min in the bioenergetic domain, at low intensities (1–2^au^ in a scale of 5) to more than 30 min at the end, at 3–4^au^, when they were autonomous and trained under self-regulated intensities. Higher intensities and cognitive challenges at the end of the program were thus possible because of the participants’ improved motor control.

(b) Nutritional counselling

The nutritional education plan was performed individually and carried out by two experienced and qualified dieticians/nutritionists. This part of the intervention was conducted once per month and was composed of an initial session of 1 h to determine the nutritional pattern of each participant and three following sessions of 30 minutes. Recommendations based on a healthy lifestyle including diet and exercise through motivational interviewing were provided following a family-based approach.

More specifically, each child attended the hospital accompanied by the family, and in each session, eating behavior was assessed and feasible objectives were planned in agreement with them (child and family)—for example, “reduce soft beverages to one can per week”. The accomplishment of objectives was then evaluated at the following visit by a new assessment of eating behavior and was discussed in a participatory way if not achieved. The nutritional counselling plan was aimed to modify unhealthy eating behavior through increasing the consumption of fresh fruits, vegetables, whole grains, legumes, fish and eggs and to decrease the intake of processed meats, sugary drinks, sweets, candies, fast food and dairy products with added sugar.

### 2.5. Statistical Analysis

Data analyses were performed using R software, version 3.3.1 (R Core Team 2016). Descriptive statistics including frequency, percentages, mean and standard deviation were employed. For normal distributions, comparisons were made using Student’s t-test; otherwise, the nonparametric Wilcoxon test was applied. Whenever feasible, an additional ANCOVA of repeated measures was conducted to account for the confounding effect of the covariates sex, age and baseline BMI z-score. Later on, in order to homogenize and analyze these changes, the effect size was calculated by means of the Cohen´s d, where the effect was considered small (d = 0.20–0.40), moderate (d = 0.50–0.70) or large (d = 0.80–2.0) according to Cohen. *p* values <0.05 were considered to be statistically significant.

## 3. Results

### 3.1. Body Composition and Biochemical Paramaters

A total of 22 children were recruited (Table 1); however, four (18.2%) of them abandoned the intervention at different points.

Table 2 shows the anthropometric data of the 18 participants (age 10.8 ± 1.6 years; 63% females) at baseline and after 16 weeks of multimodal intervention. Z-scores were significantly greater at baseline compared with post-intervention measurements. Before the intervention, children presented z BMI above 3; afterwards, a drop to 2.7 was achieved (*p* < 0.001). MUAC (*p* < 0.01), TRI (*p* < 0.001) and SUB (*p* < 0.01) skinfolds were reduced by around 0.5 points. WHtR was also reduced (*p* < 0.001). Body fat of participants was 2.4 and 4% lower depending on the methodology (*p* < 0.001). Moreover, a decrease of 2.7% in trunk fat was also detected (*p* < 0.01). None of the covariates (baseline z BMI, sex and age) influenced these results except for z WC, where the reduction of 0.5 points became statistically significant only when controlling for sex and baseline z BMI (*p* < 0.05 for both covariates independently).

With regard to the biochemical parameters (Table 3), children had statistically significantly lower levels of LDL-C (*p* < 0.05), GGT (*p* < 0.01), ALT (*p* < 0.05), CRP (*p* < 0.05) and HOMA-IR (*p* < 0.05) after attending the intervention. There was no influence of either of the covariates.

### 3.2. Physical Function

Table 4 shows pre-post changes in neuromuscular, cardiovascular fitness and fundamental movement patterns. Children had statistically significantly higher scores on the mPACER test (*p* = 0.003), FMS_7 (*p* < 0.001) and FMS_4 (*p* < 0.001), obtaining a higher total and partial score.

## 4. Discussion

As a main finding of this pilot study, 16 weeks of a multimodal intervention comprising two hours a week of periodized and enjoyable, supervised multicomponent exercise, together with professional nutritional counselling, has been enough to improve body composition and biochemical markers, as well as cardiovascular fitness and psychomotor skills (fundamental motor patterns) in a group of obese children.

Specifically, there were significant improvements in body size and body composition, as reflected by the changes in z BMI, z MUAC, WHtR, z TRI and z SUB skinfolds, body-fat Slaughter equation and DXA body fat% and trunk fat%. Results showed also improvements in several biochemical parameters such as LDL-C, hepatic enzymes GGT and ALT, the HOMA-IR and the CRP inflammatory marker. Among these, HOMA-IR index is remarkable since it is considered a marker of insulin resistance, so its decrease probably indicates enhanced metabolic behavior, which may help to reduce the incidence of future comorbidities. In previous studies, we already observed that HOMA index is a cardiovascular risk factor that increases as obesity worsens, which emphasizes the importance of prevention [45,46]. Moreover, the measurement of the effect of interventions on clinical correlations associated with obesity has been suggested [47]. Previous authors have demonstrated that reductions of 0.25–0.50 SD in z BMI are associated with decreased levels of triglycerides, HOMA-IR and TGA/HDL-C ratio as well as an increase in HDL-C [48]. In this pilot study, 0.30 SD drop in z BMI was achieved in 16 weeks of combined physical activity and nutritional counselling and was already related to improvements in biochemical parameters. Similar to our study, other interventions have demonstrated a reduction in HOMA-IR [16,49], with an average decrease of −0.61 units, as shown by a recent meta-analysis [9].

Despite the short duration of this intervention, there is an outstanding reduction in the obesity scores and a possible reduction in the low-graded persistent inflammation (CRP) associated with this disorder. In the European study HELENA (Health Lifestyle in Europe by Nutrition in Adolescence), several inflammatory biomarkers measured in adolescents from nine countries showed that HOMA-IR and CRP together with complement C3 exhibited the highest levels of BMI and fat index [50,51]. These results were confirmed for a large sample of children, supporting the inflammatory state related to BMI. Our intervention also seems to mitigate the inflammatory effects of obesity, as shown by the decrease in CRP, in line with previous research [16]. Nevertheless, recent meta-analyses of clinical trials show a trend towards reduction in CRP related to physical activity intervention, but not significant changes [52], so more studies and follow-up of participants will be needed to confirm this finding.

Interventions aimed at reducing obesity in childhood are common in the scientific literature, but comparisons among studies are difficult to perform due to the multiple differences in terms of duration, intensity or outcome variables and nutritional criteria, among others. In addition, this literature often reports low attendance and little participation, with drop out related to injuries, lack of motivation, little self-confidence, perceived and/or real physical incompetence, etc. [18,23,31]. These psychophysiological and motor factors are most times related to an initial low fitness level and lack of previous positive exercise experiences in the obese pediatric population [18,23,31], which may be discouraging and demotivating for both the children and the team of professionals working with them.

A meta-analysis including after-school interventions displayed a general statistically significant reduction in the BMI of children [53]. Since anthropometric measurements are often inaccurate and errors may hamper the detection of changes in terms of body composition, we included the application of more accurate assessment techniques such as the recommended DXA scanning [54]. This is non-invasive and has the sensitivity to determine changes in body composition that other techniques do not have [55], becoming useful for the follow up of various endocrine, metabolic and nutritional diseases and for monitoring the effects of their treatments. We would like to point out that the anthropometry of this study was carried out by the same properly trained observers. As a consequence, the significant reduction in body fat was evidenced by both anthropometry as well as by absorptiometry techniques. In agreement with our findings, it has been reported that exercise significantly reduced body fat in obese children of around 12 years of age, particularly after interventions including behavioral dietary restriction [47]. In fact, exercise has been associated with a reduction in visceral, subcutaneous and intrahepatic fat [56].

With regard to central fat, its reduction is key to improve the cardiometabolic status of obese children, as abdominal fat has been associated with insulin resistance in this population [57]. WHtR has been proposed as a useful indicator of cardiovascular risk [58] and visceral adiposity prediction, even though it is not the gold-standard [57]. In this way, a significant reduction in abdominal fat assessed by DXA and WHtR was obtained in our study, which might be associated with the improvements in the biochemical parameters. Along with body fat and trunk fat reduction, the improvement in HOMA-IR is noteworthy, since insulin resistance is considered the main factor in the development of other obesity associated comorbidities [5], as already mentioned.

In general, the duration of these multimodal interventions ranges between 6 and 30 weeks, with most of them lasting around 12 to 20 weeks [47], but our pilot study took only 120 min/week for 16 weeks (14 weeks of training). Expert committees recommend exercise training doses close to 210–360 min/week [10,12,18,23,59,60]. However, programs including more than 200 min/week of physical activity are rarely found in childhood obesity literature [47]. Our outcomes support the notion that lower doses of exercise than those recommended are also able to induce significant improvements of anthropometric parameters, despite the benefits of additional volumes of exercise that might be expected. Moreover, given the chronic character of obesity, the long-term follow-up of the outcomes analyzed would be advisable. It would be of major relevance if these improvements could be preserved in the long term, as it has been demonstrated that insulin resistance at the beginning of puberty is associated with insulin resistance at the beginning of adulthood [61] and higher blood pressure, dyslipidemia, vascular stiffness and thickness [62].

Notably, resistance training or any other neuromuscular exercise proposal becomes metabolic by modifying some training parameters, such as increasing the intensity and the muscle mass in the exercises and/or reducing the recovery laps within sets and series. Our multicomponent exercise complex approach achieved its goals by maintaining moderate to vigorous intensities and large muscle mass participation throughout the 60 min period, thanks to the combination of neuromuscular exercises including strength, balance and mobility postural control exercises, alternated with resistance training with challenging circuits, fighting games and other fun-type skill-learning physical activities, even with some sets of mini-sport games. Previous multimodal interventions from Morano et al. [31,63], based on mini sports games and motor control exercises, also aiming to reduce weight and improve the basic motor competences and quality of live in obese children, already promoted fun and enjoyable experiences and confirmed that these are good strategies to adhere obese children to physical activity. Children need to have fun and play with their peers and not follow strict rules or practice alone [64]. We have not measured any psychological item or long-term adherence to exercise, which is a limitation of our study, but enjoyable interventions have been shown to promote long-term motivation and engagement with exercise and physical activity [65], which we intended.

It has been already stated that these mixed exercise proposals (strength and aerobic integrated in continuous protocols) induce improvements in body composition and cardiovascular health similar to aerobic exercise alone [8,10,12,18]. They have shown a slightly lower impact on fat reduction as compared to aerobics in overweight and obese adults, but not in children, where they equate [8]. This might shed light on the significant changes that we observed in the short term. Populations with metabolic syndrome, overweight and type II diabetes benefit from these multicomponent mixed approaches [12,18], because, while the gain in muscle mass typical of resistance training increases the basal caloric expenditure and concomitant glucose regulation, with no changes in insulin resistance at the muscular level, aerobic programs—especially due to the moderate–vigorous intensity—improve glucose absorption, associated with increased insulin activity and regardless of muscle mass or aerobic capacity [12,18]. Our multicomponent exercise program aimed to combine different exercises and training objectives, built up under the temporal and metabolic structure of continuous protocols of moderate to vigorous intensity. This metabolic approach to exercise has already worked in overweight children [8,26,66]. Furthermore, a recent systematic review [67] points out that improving fundamental movement skills could break the vicious cycle of childhood obesity, in which obese children do not practice physical activity due to a lack of physical literacy, which feeds back to itself. The multicomponent exercise program was diary tailored to the children, supervised by professionals in sports sciences and periodized as a physical conditioning program, combining all the above ingredients with the main principles of exercise training. The addition of nutritional counselling and the regular presence of the nutritional professionals in the sport facilities might have also be a key component of our short-term significant benefits.

On the other hand, we also introduced and interspersed some very short burst and controlled speed activities, as well as sport games, dual tasks, motor and cognitive conditional challenges and other fun activities, because they are typical of the children’s movement patterns [8,18,68]. It is crucial to develop enjoyable programs [10,31,63,66]. It is equally essential to influence the intrinsic motivation for exercise and self-care to be successful and avoid the regain of weight in this population [22,23,31,63,66]. In line with these authors, our individualized and tailored multimodal design, and the team work in this multidisciplinary approach, may compensate for the lack of a strict reduction of calories or the low volume and frequency of training as compared to the obese exercise prescription guidelines, helping these children to improve their overall health without too much time investment or significant personal sacrifices. Furthermore, the second mesocycle in the intervention (weeks 9 to 14) also increased gradually the participants’ autonomy, making them value their own decisions and capacities, thus promoting the enjoyment of physical activity and exercise without the trainer and adults’ rules.

Our participants had good cardiovascular fitness before the intervention, thus helping to obtain our good results. Baseline VO_2_ max obtained from Leger, et al. [69] equation was 37.83 mL/kg/min, which, compared to normative data for obese girls and boys [70], is above the percentile 25 that is considered as a metabolic risk. In addition, comparing the broad jump with other studies, our baseline outcomes were higher than the study by Morano, et al. [63] (124 cm vs. 97 cm—boys and 108 cm—girls) and Pienaar, et al. [71], whose participants reached 126 cm after the intervention (similar to our study). Handgrip strength also matched the reference data [72]. Regarding the fundamental patterns and the motor competence, to our knowledge, this is the first time that the FMS has been used to follow an intervention in obese children. Our participants had low pre-training values, but they reached a 10.9 score after the multicomponent training program. Similar to the previous outcomes, this value is high for obese children (score = 9) [43], but it is far from the 14-points in normal-weight children [43,73]. This baseline fitness level highlights the importance of early interventions in the obese and overweight population, since children are still able to participate and enjoy physical exercise and wellness with little difficulty. Better baseline physical fitness may have also helped to obtain the large effect size in the mPACER test and the FMS (4 and 7), thus favoring the continuity of physical activity in the future, a concomitant important target for any multimodal educative intervention. Additionally, the improvement in the FMS points out enhanced motor control, which has been shown to contribute to better and larger participation in future physical activity [67].

Neither lower limb strength nor handgrip benefited from our multicomponent program, despite the improvements in cardiovascular fitness and FMS. Pienaar, et al. [71] also failed to improve the broad jump in a group of children with 11 years after 13 weeks of intervention, suggesting the need for longer or more specific training programs for strength in this population. For example, 3 years for the lower limb strength in a similar sample (obese children between 8 and 11 years) [74].

To conclude, despite our high rates of adherence, several obese children could not participate (mainly due to personal or family time schedule problems or transport difficulties but also due to parenteral disagreement with regard to obtaining help through the program and even social dysfunction). Indeed, a few children displayed refusal to exercise. Future interventions should make the effort to help and motivate these children—for example, by inviting them to attend some sessions to see their peers, looking for inspiration—and help their families—for example, by taking these supervised and coordinated multimodal programs to the schools, healthcare centers or closer sports facilities, supported by governmental health policies.

This study is not without limitations. On the one hand, the sample size is small and limits the power of our good results. In addition, there is not a control group who did not exercise or follow a nutritional counselling alone, but this was intentional from an ethical point of view because we aimed to benefit the children; there was wide evidence about the benefits of physical exercise for pediatric obesity, and there were no financial aids to support larger samples. This is why we developed a pilot study. On the other hand, we chose not to present some pre-post cardiovascular data which were collected at the hospital and would add valuable information to our results.

## 5. Conclusions

Despite these limitations the results presented in this work confirm the feasibility and benefits of applying multimodal intervention including multicomponent exercise and nutritional counselling supervised by specialists in physical exercise in conjunction with pediatric gastroenterologists and trained nutritionists.

Weight management strategies based on lifestyle modifications and health educational multimodal interventions should be considered a first-line therapy for childhood obesity, since other treatments such as very low-calorie diets, pharmacotherapy or bariatric surgery are not generally accepted as safe [75].

Multimodal strategies and multidisciplinary collaboration will improve and benefit from further studies developed to confirm and improve our preliminary results. Further interventions should include some psychological and quality of life items, as well as the follow-up of all these variables, to better understand its long-term impact. Assessing the long-term adherence to exercise and proper nutritional habits is also important.

Therefore, the proposed program may suppose a useful tool to reduce the burden and prevalence of childhood obesity and its associated comorbidities.

## Figures and Tables

**Table 1:** Characteristics of the study population.

Participants (n = 22)	Baseline	
Age (years)	10.9 (1.7) (Range 8–14)	
Gender (%)	Female	n = 13 (59%)
	Male	n = 9 (41%)
Tanner stage (%)	Prepubertal	n = 11 (50%)
	Pubertal	n = 11 (50%)

**Table 2:** Evolution of the anthropometric measurements expressed as mean (SD).

	Baseline	Post-Intervention	*p* Value
z BMI	3 (0.4)	2.7 (0.4)	<0.001
z WC	6.8 (1.4)	6.3 (1.2)	<0.05 ^a^
z MUAC	3.4 (1)	3 (0.9)	<0.01
z TRI	3 (0.8)	2.4 (0.7)	<0.001
z SUB	3.7 (0.8)	3.2 (1.1)	<0.01
WHtR	0.64 (0.04)	0.61 (0.03)	<0.001
Body fat (Slaughter, %)	44.6 (6.9)	40.7 (5.5)	<0.001 ^b^
Body fat (DXA, %)	46 (4.4)	43.6 (4.6)	<0.001
Trunk fat (DXA, %)	45.3 (4.4)	42.6 (5.1)	<0.01

^a^ ANCOVA repeated measures, controlling for the covariate sex, as well as for baseline z BMI; ^b^ Wilcoxon test. Z-score is related to cut-off points by age [32]. BMI: body mass index; WC: waist circumference; MUAC: mid upper arm circumference; TRI: tricipital skinfold; SUB: subscapular skinfold; WHtR: waist-to-height ratio; Slaughter: equation with tricipital and subscapular skinfolds [35]; DXA: dual energy X-ray absorptiometry.

**Table 3:** Evolution of the biochemical parameters expressed as mean (SD).

	Baseline	Post-Intervention	*p* Value	*d*
Cholesterol (mg/dL) (*n* = 17)	165 (34)	157 (31)	Ns	−0.25
HDL-C (mg/dL) (*n* = 17)	50 (10)	49 (14)	Ns ^a^	−0.08
LDL-C (mg/dL) (*n* = 17)	108 (27)	100 (24)	<0.05	−0.31
Triglycerides (mg/dL) (*n* = 17)	79 (36)	84 (47)	Ns	0.12
Uric acid (mg/dL) (*n* = 16)	4.9 (1.2)	4.9 (1.5)	Ns	0
GGT (U/L) (*n* = 15)	23 (12)	18 (8.2)	<0.01	−0.49
ALT (U/L) (*n* = 18)	25 (12)	21 (9)	<0.05	−0.38
Glycated hemoglobin (%) (*n* = 14)	5.3 (0.1)	5.3 (0.2)	Ns	0
C-reactive protein (mg/dL) (*n* = 16)	4 (3.2)	2.2 (1.9)	<0.05	−0.68
Glucose (mg/dL) (*n* = 17)	95 (6)	94 (6)	Ns	−0.17
Insulin (µU/mL) (*n* = 17)	24 (17)	20 (8)	Ns ^a^	−0.3
HOMA-IR (*n* = 17)	5.6 (3.9)	4.6 (2.1)	<0.05 ^a^	−0.32

^a^ Wilcoxon test. *d*: Cohen effect size; Ns: not significant; HDL-C: high density lipoprotein cholesterol; LDL-C: low density lipoprotein cholesterol; GGT: gamma-glutamyltransferase; ALT: alanine aminotransferase; HOMA-IR: homeostatic model assessment of insulin resistance.

**Table 4:** Changes in physical function expressed as mean (SD).

	Baseline	Post-Intervention	*p*-Value	*d*
Handgrip_right (kg)	23.13 (4.98)	23.89 (5.47)	Ns	0.15
Handgrip_left (kg)	20.44 (5.54)	20.36 (7)	Ns	−0.01
Broad jump (cm) (*n* = 17)	124.73 (16.58)	126.5 (13.81)	Ns	0.12
mPACER test (laps) (*n* = 16)	18.81 (3.6)	23.81 (6.35)	0.003	0.97
mPACER test (km/h) (*n* = 16)	8.47 (0.62)	8.94 (0.4)	0.006	0.9
FMS_7 (*n* = 17)	8.76 (1.82)	10.7 (2.28)	<0.001	0.94
FMS_4 (*n* = 17)	5.35 (1.58)	6.94 (1.44)	<0.001	1.05

*d*: Cohen effect size; Ns: not significant; mPACER: cardiovascular fitness; FMS_7: functional movement screen (7 exercises); FMS_4: functional movement screen (4 exercises).
